# 

**Published:** 1890-09

**Authors:** 


### CONTENTS:

PAGE

ORIGINAL ARTICLES:

Rabies and Strongylus Tetracanthus as a Coincidence in the Horse. By G. W. Butler, V. S. . . . 483

Serous Cyst in Superior Maxillary Sinus. By J. H. Frink, V. S. . . . 491

Age of the Horse, Ox, Dog, and other Domesticated Animals. By R. S. Huidekoper, M.D., Veterinarian . . . 493

Hydrops Uteri as a Sequel to Imperforate Vagina. By William G. Martin, V. S. . . . 510

EDITORIAL DEPARTMENT:

United States Veterinary Medical Association . . . 512

Pennsylvania State Veterinary Medical Association . . . 513

CASES, SELECTIONS:

Dehorning Cattle . . . 513

Veterinary Jurisprudence. The Dehorning of Cattle—Test Prosecution at Kells . . . 516

The Operative Treatment of Roaring . . . 521

NECROLOGY:

William Raeburn Jenkins . . . 524

SOCIETY PROCEEDINGS:

Ohio State Veterinary Medical Association . . . 525

New York State Veterinary Medical Society . . . 526

Veterinary Medical Association of New Jersey . . . 527

VETERINARY COLLEGE NOTES:

American Veterinary College (New York) . . . 528

New York College of Veterinary Surgeons . . . 528

Baltimore School of Veterinary Science . . . 528

Faculty of Comparative Medicine, McGill University. Montreal . . . 528

Chicago Veterinary College . . . 529

Franklin County (Pennsylvania) Veterinary Society . . . 529

CORRESPONDENCE:

Open Letter . . . 530

REVIEW DEPARTMENT . . . 533

BOOKS AND PAMPHLETS RECEIVED . . . 534

